# Task model-specific operator skill assessment in routine fetal ultrasound scanning

**DOI:** 10.1007/s11548-022-02642-y

**Published:** 2022-05-12

**Authors:** Yipei Wang, Qianye Yang, Lior Drukker, Aris Papageorghiou, Yipeng Hu, J. Alison Noble

**Affiliations:** 1 Institute of Biomedical Engineering, Department of Engineering Science, University of Oxford, Oxford, UK; 2 Department of Medical Physics and Biomedical Engineering, University College London, London, UK; 3 Nuffield Department of Women’s and Reproductive Health, University of Oxford, Oxford, UK

**Keywords:** Skill assessment, Ultrasound, Fetal ultrasound, Deep learning

## Abstract

**Purpose:**

For highly operator-dependent ultrasound scanning, skill assessment approaches evaluate operator competence given available data, such as acquired images and tracked probe movement. Operator skill level can be quantified by the completeness, speed, and precision of performing a clinical task, such as biometry. Such clinical tasks are increasingly becoming assisted or even replaced by automated machine learning models. In addition to measurement, operators need to be competent at the upstream task of acquiring images of sufficient quality. To provide computer assistance for this task requires a new definition of skill.

**Methods:**

This paper focuses on the task of selecting ultrasound frames for biometry, for which operator skill is assessed by quantifying how well the tasks are performed with neural network-based frame classifiers. We first develop a frame classification model for each biometry task, using a novel label-efficient training strategy. Once these *task models* are trained, we propose a second task model-specific network to predict two skill assessment scores, based on the probability of identifying positive frames and accuracy of model classification.

**Results:**

We present comprehensive results to demonstrate the efficacy of both the frame-classification and skill-assessment networks, using clinically acquired data from two biometry tasks for a total of 139 subjects, and compare the proposed skill assessment with metrics of operator experience.

**Conclusion:**

Task model-specific skill assessment is feasible and can be predicted by the proposed neural networks, which provide objective assessment that is a stronger indicator of task model performance, compared to existing skill assessment methods.

**Supplementary Information:**

The online version contains supplementary material available at 10.1007/s11548-022-02642-y.

## Introduction

Skill assessment of imaging specialists has long been established as an essential tool in training, continuing education, and clinical service auditing and improvement [1]. Ultrasound imaging is a particularly hard skill to learn and is known to be operator dependent. Ultrasound skill needs to capture both the ability to read and to take accurate diagnostic measurements on images (as in radiology) but also the ability to capture diagnostic images (unlike in radiology). Traditional quantitative metrics such as time-to-completion and clinical outcome have been used to provide quantitative evidence to inform, design, and deliver skill assessment methods [2, 3] but are considered simplistic.

Building on recent advances in machine learning in imaging, especially in deep learning, recent efforts have proposed neural network-based automatic skill assessment approaches [4–8]. Most of these learning-based methods automate skill assessment by predicting the skill level from available intra-procedure data, including imaging [4], motion [5, 6], or a combination of both [7]. The training labels that represent skill level include operator experience, such as length of practice [6] and other competence indicators such as different clinical roles [8]. Machine learning algorithms have also been proposed to measure task-specific image quality [9] that may be partially indicative of skill assessment and directly relevant to the clinical tasks to hand.

Standard plane selection [10], gestational age regression [11], and direct biometry estimation [12] are common clinical fetal ultrasound imaging tasks that have been considered for automation by machine learning approaches. Indeed, sufficient progress has been made for some solutions to appear in commercial systems. In this paper, we consider the impact that the introduction of such *task models* has on the skills required to acquire, interpret, and report ultrasound images and the assessment of the “new” skills. For example, a frame classifier may indicate that sufficiently accurate biometry can be reliably measured on an ultrasound frame deemed “positive”, while a “negative” frame potentially suggests the need for image re-acquisition. Such a classification model may relax the definition of a standard plane and subsequently alter the skill required to acquire frames amenable to the classifier.

In this paper, we focus on two established clinical tasks in fetal ultrasound examination, measuring the head circumference and the abdomen circumference on ultrasound video frames. Firstly, for each task, a frame classification model is trained using a novel label-efficient approach that utilises mixed manual and image-similarity-based surrogate labels from a high volume of ultrasound video frames. Secondly, we propose to measure how well these task models perform for assessing operator skill level, then develop new neural networks to predict these skill levels based on real-time ultrasound images and synchronised probe motion data. These *skill assessment predictors* are trained to quantify two proposed criteria that are directly informed by frame classification performance. In addition to these methodological developments, our contributions in this paper include a set of systematic results showing the efficacy of both task models and skill assessment predictors, together with detailed analysis with ablation studies evaluating the importance of a selection of network hyperparameters, training strategy options, varying input data combinations, and different skill level definitions.

**Fig. 1:**
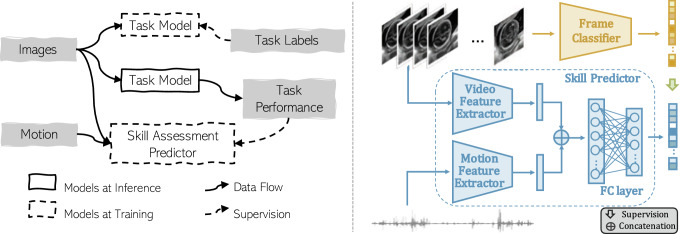
Overview of the task model-specific skill assessment framework

## Method

An overview of the proposed task model-specific skill assessment framework is shown in Fig. 1. Let $J^{i}$ be the available number of ultrasound video frames from *i*th subject. A total of $\sum _{i}^{I}J^{i}$ video frames $\{\{v_i^j\vert j=1,\ldots ,J^i\}\vert i=1,\ldots ,I\}$, sampled from a set of *I* video clips $V_{i}=\{v_{i}^{j}\vert j=1,\ldots ,J^{i}\}$, provide the training input for the task model which performs a clinical task of interest, developed in section “Frame classification networks”. The task labels $\{\{g_i^j\vert j=1,\ldots ,J^{i}\}\vert i=1,\ldots ,I\}$ are application dependent, such as the suitability of given frames for fetal biometry (see “Label-efficient mixed ground-truth generation” section). The ultrasound video clips $\{V_i\}$ are then combined with synchronised probe motion data $\{M_i$}, for training a second neural network for skill assessment, supervised by a measure of task performance $\{f_\mathrm{skill}({\hat{G}}_i)\vert i=1,\ldots ,I\}$, based on the task model predictions ${\hat{G}}_i=\{{\hat{G}}_{i}^{j}\vert j=1,\ldots ,J^i\}$ at inference. This skill assessment predictor is described in section “Skill assessment predictors”, with two proposed definitions of task performance $f_\mathrm{skill}(\cdot )$.

### Task models

#### Frame classification networks

Without loss of generality, we consider an end clinical task being a binary classification. Specifically in this work, the tasks are to classify whether a sufficiently accurate measurement can be obtained on individual frames in the ultrasound video. For each clinical task described in “Clinical tasks and data acquisition” section, a deep neural network $f_\mathrm{task}({\mathcal {V}};\theta ):{\mathcal {V}} \rightarrow {\mathcal {G}}$, with learnable parameters $\theta$, is trained to predict the probability ${\hat{g}}_i^j=f_\mathrm{task}({\mathcal {V}};{\hat{\theta }})$ of a positive class for each input frame ${\mathcal {V}}=v_i^j$. Optimising parameters ${\hat{\theta }}$ requires a ground-truth label ${\mathcal {G}}=g_i^j$ for every input frame $v_i^j$, during the supervised network training. The details of network training are summarised in “Model implementation and training” section.

#### Label-efficient mixed ground-truth generation

In the routine second trimester fetal ultrasound examination, the fetal head circumference (HC) and abdominal circumference (AC) are measured on biometry planes selected manually by operators following the standard plane definitions detailed in widely-adopted guidelines [13]. In order to train the frame classification networks, labels are required for all image frames in the entire ultrasound video. We therefore propose a practical approach to utilise a limited number of manually annotated labels from each subject, then use them to regress the binary labels on the unlabelled frames using a set of image similarity measures between a given frame and the one selected as the diagnostic plane $v^{d.p.}_{i}$ for the *i*th subject. Five image similarity metrics were adopted, cosine similarity in feature space [14], mean squared error, cross correlation [15], mutual information, and structural similarity index [16]. To calculate the cosine similarity, an in-house ultrasound-based pre-trained model was used to extract image features.

For any given frame $v_i^j$, the percentage relative error is defined directly between the biometry $f_\mathrm{biometry}(v_i^j)$ obtained on the frame and that from the diagnostic plane of the same *i*th subject: ${\delta }_i^j = \frac{f_\mathrm{biometry}(v_i^j)}{f_\mathrm{biometry}(v^{d.p.}_{i})} \times 100\%$. Then, the fitted linear model ${\hat{\delta }}_i^j=f_\mathrm{linear}({\mathcal {V}}=v_i^j; v^{d.p.}_{i})$ predicts an estimation ${\hat{\delta }}_i^j$ of the percentage relative error. Thus, the surrogate binary label $g_i^j$ for an unlabelled frame $v_i^j$ can be generated using a minimum threshold $\delta _\mathrm{min}$ on the estimated ${\hat{\delta }}_i^j$, such that: $$g_i^j=\left\{ \begin{matrix} 0, &{} {\hat{\delta }}_i^j>\delta _\mathrm{min} \\ 1, &{} \mathrm{otherwise} \\ \end{matrix} \right. $$. The threshold $\delta _\mathrm{min}$ has a direct clinical interpretation, the precision of target biometry, which is of significance in controlling the quality of the mixed labels.

### Skill assessment predictors

The skill assessment predictor $f_\mathrm{assess}({\mathcal {V}},{\mathcal {M}};\omega ):{\mathcal {V}},{\mathcal {M}}\rightarrow {\mathcal {S}}$ is a multi-modal neural network with trainable parameters $\omega$, which takes $\tau$ consecutive frames ${\mathcal {V}}=\{v_i^j\vert j=n,\ldots ,n+\tau -1\}$ as well as the corresponding probe motion segments ${\mathcal {M}}=\{m_i^j\vert j=n,\ldots ,n+\tau -1\}$ and predicts a task performance score ${\mathcal {S}}={\hat{S}}_i^{n}$ during this time interval, where $n=1,\ldots ,J^i-\tau +1$ and each video sequence forms $J^i-\tau +1$ training data. The network is optimised with the mean-squared-error (MSE) loss $L = {\mathbf {E}}_{i,n}[(S_i^n-{\hat{S}}_i^n)^2]$, where ${\mathbf {E}}_{i,n}[\cdot ]$ is the expectation over all training data and $S_i^n=f_\mathrm{skill}(\{{\hat{g}}_i^j\vert j=n,\ldots ,n+\tau -1\})$ is the supervising task performance score obtained from the frame classification networks, and is considered as an estimate of $f_\mathrm{skill}({\hat{G}}_i)$, for the *i*th subject at the *n*th available time point. In the following subsections, two types of task performance score are introduced for operator skill assessment.

#### Expected positive task prediction

For both tasks considered in this paper, positive frames enable successful biometric measurement. Therefore, we first investigate a task performance score defined as the expected value of probability ${\mathbf {E}}[{\hat{g}}_i^j]$, for the positive class predicted by the task models. Namely, for the *i*th subject at the *n*th time point:

1 $$\begin{aligned} f_\mathrm{skill}^\mathrm{pos}(\{{\hat{g}}_i^{j}\};n)=\frac{1}{\tau }\sum _{j=n}^{n+\tau -1}{{\hat{g}}_i^j}, \end{aligned}$$

#### Expected task prediction accuracy

A second skill assessment criterion determines how accurate the task model predicts both a positive and a negative class, rather than the probability of obtaining a positive class. Correct prediction of a negative class is therefore considered as important as the correct prediction of a positive frame. This definition takes into account both risks with positive and negative frames being incorrectly classified, corresponding to Types 1 and 2 errors, rather than favouring a high positive rate only (as Eq. 1 could). Let $\mathrm{TP}_i^n(c)$ and $\mathrm{TN}_i^n(c)$ represent the number of true positive and true negative frames respectively. The second task performance score for assessing skill level is then defined in terms of the binary classification accuracy, at the *n*th time point:

2 $$\begin{aligned} f_\mathrm{skill}^\mathrm{acc}(\{{\hat{g}}_i^{j}\};n,c)=\frac{\mathrm{TP}_i^n(c)+\mathrm{TN}_i^n(c)}{\tau }, \end{aligned}$$

where *c* is a pre-defined cut-off on the task model-predicted class probability.

## Experiments

### Clinical tasks and data acquisition

Ultrasound video and probe motion data used in this study were acquired as part of the PULSE study, approved by the UK Research Ethics Committee (reference 18/WS/0051) [17]. Scans were performed from June 2018 to February 2020 at the Oxford University Hospitals NHS Foundation Trust, by sonographers and fetal medicine doctors (collectively referred to as operators in this paper) using a Voluson E8 scanner (GE Healthcare, USA), with curvilinear (C2-9-D, C1-5-D) and 3D/4D (RAB6-D) probes. Written informed consent was given by all participating operators and pregnant women. To acquire probe motion data, an inertial measurement unit (Next Generation IMU (NGIMU), x-io Technologies Limited, Bristol, UK) was fixed at the same probe position during all scans. The raw motion signal was recorded at 400 Hz and then down-sampled with the timestamp-synchronised ultrasound video frames, losslessly frame-grabbed and recorded at 30 Hz.

A subset of the second trimester scans from the PULSE dataset was used, with manual labels of head circumference and abdominal circumference obtained using the available user interface on the scanner. Up to 12 s of video before the operator stops scanning for measurement was extracted, which resulted in an average of 10.61 s of consecutive frames. A total of 294 clips, for scanning fetal head and abdomen which have consistent zoom factors and a length of at least 6 s, formed the dataset used in this study, for the purpose of controlled experiments. In summary, up to 84 clips with 27k video frames from each of the 11 individual sonographers were included in this study. Further details of the data contributions with respect to different operators are included in the supplementary materials.

*Head circumference (HC) measurement* is a routine clinical task that indicates a number of clinical anomaly conditions and is also used for gestational age estimation. HC measurement is typically taken on a manually selected diagnostic plane. In this experiment, we investigate a frame classification model to assist this task by differentiating individual ultrasound image frames, from which the measured HC is sufficiently accurate, using a total of 146 HC video clips. To generate the task model labels for this task, 345 frames from randomly sampled 22 clips were annotated manually. The circumferences were calculated on fitted ellipses, in an “outer-to-outer” manner [18].

*Abdominal circumference (AC) measurement* is another important biometry that is correlated to fetal growth parameters, such as weight and gestational age. Similar to the frame classifier defined for assisting the HC measurement task, experiments were designed to examine a frame classification model for accurate AC reporting. From 19 out of 149 available AC clips, 298 frames were annotated manually for generating the mixed labels.

### Model implementation and training

The linear regression models for training label generation were fitted on 266 and 228 images from 17 and 14 clips and were tested on 79 and 70 holdout test images, for the HC and AC tasks, respectively.

All ultrasound images used in this work were resized to $224 \times 288$ pixels and normalised to zero-mean and unit-variance. A VGG-based network [19] was adopted with the same implementation details as the task models for the purpose of a reference quality benchmark in this work. For skill assessment prediction, a VGG16 network [20] was adopted for extracting features from a stack of consecutive ultrasound images, with a 1D ResNet18 network [21] for extracting features from 1D probe motion signals, here, the three-axis angular velocities formed the three-channel 1D input. The input window size is referring to the number of input images and the length of the synchronised probe motion signals. Features from the two branches were concatenated via adaptive pooling, resulting in a feature vector with a length of 32, and this feature vector was fed into linear layers which predict the task performance score, as illustrated in Fig. 1. All networks were implemented in PyTorch and trained with a mini-batch size of 64 on the NVIDIA Quadro GV100 GPUs using an Adam optimiser with a learning rate of $10^{-4}$. The task models and skill assessment predictors were trained with cross-entropy and MSE losses, for 50 and 700 epochs, respectively.

### Model evaluation and ablation studies

Task models were evaluated on the acquired ultrasound video dataset as detailed in “Clinical tasks and data acquisition” section. The dataset was randomly partitioned into a development set and a holdout test set, without any operators or subjects in both sets. This partition was repeated to form the A and B “splits” for the HC task and the C and D “splits” for the AC task. Further details of dataset split are provided in the supplementary material. A further 85:15 random split of the development set resulted in a training set and a validation set during developing the models, while models were trained with different hyperparameters and those with the highest class-balanced accuracy on the validation set were selected. The results reported in this paper are based on these selected models tested on the holdout test set. All models were trained and tested using the generated surrogate labels. The reason to only test the surrogate labels is that, in real-world ultrasound scanning, we do not expect the sonographers to make manual annotations on frames that are not selected as the diagnostic plane. Hyperparameters investigated in this work include learning rate, mini-batch size and, for training the task models, multiple consecutive frames for contextualising the input (a five-gram context) were also tested. A ResNet18 network [21] for extracting ultrasound image features was also tested and the results were not found significantly different to those using VGG16, therefore they are included in supplementary material.

The accuracy, sensitivity, and specificity were computed for ablation studies to evaluate the task models, with varying precision requirements, $\delta _\mathrm{min} \in \{1\%,2\%,3\%,4\% \}$, and different cut-off values on predicted class probabilities, such that the resulting specificity values are controlled at 0.8 and 0.9 as examples of clinically acceptable false positive rates.

Skill assessment predictors were tuned on the validation set and those that achieved the lowest root mean square error (RMSE) were used to report results on the test set. Two metrics were computed to compare ablation studies on varying window size $\tau$, for each of the development-test splitting strategies and for each of the HC and AC tasks, (1) the RMSE between the predicted and the task model-generated performance scores and (2) the Pearson correlation coefficient (PCC) between them. The impact from different types of input data types was also compared, by using either the video data or motion data alone for training and testing the skill assessment predictors.

## Results

### Quality of the mixed label generation

The mixed label generator $f_\mathrm{linear}$ described in Section “Label-efficient mixed ground-truth generation” was tested on an independently sampled subset with manual labels as ground truth. The predicted relative percentage error ${\hat{\delta }}$ achieved MSE values of $0.00076\pm 0.00046$ and $0.00084\pm 0.00048$, on the HC and AC datasets, respectively. Of note, this error is with a normalised range of [0, 1], therefore the absolute MSE is reported. Further visualisation results of the mixed label generator performance are provided in the supplementary materials.

### The head circumference task

#### Task model performance

For both datasets A and B, the task model accuracy for the HC task generally increases as the allowed percentage error $\delta _\mathrm{min}$ increases, with consistent sensitivity and specificity values observed at a default cut-off value of 0.5. The performance of the task model with HC dataset is summarised in Table 1.

**Table 1:** Task model performance for different data splits as $\delta _\mathrm{min}$ varies

Task	Split	$\delta _\mathrm{min}$	Accuracy	Sensitivity	Specificity	Task	Split	$\delta _\mathrm{min}$	Accuracy	Sensitivity	Specificity
HC	A	0.01	0.58	0.79	0.52	AC	C	0.01	0.80	0.38	0.84
		0.02	0.74	0.80	0.64			0.02	0.71	0.62	0.75
		0.03	0.73	0.71	0.82			0.03	0.71	0.58	0.82
		0.04	0.89	0.90	0.66			0.04	0.64	0.59	0.76
	B	0.01	0.68	0.34	0.82		D	0.01	0.60	0.57	0.60
		0.02	0.67	0.60	0.80			0.02	0.73	0.69	0.75
		0.03	0.77	0.79	0.66			0.03	0.77	0.79	0.74
		0.04	0.85	0.87	0.56			0.04	0.65	0.61	0.77

**Table 2:** Ablation study results for different input data modalities at $\delta _\mathrm{min}=0.04$

Criterion	Data modality	RMSE	PCC	Criterion	Data modality	RMSE	PCC
$f_\mathrm{skill}^\mathrm{acc}$	Both	0.164 ± 0.189	− 0.176 ± 0.201	$f_\mathrm{skill}^\mathrm{pos}$	Both	0.171 ± 0.116	0.212 ± 0.257
	Motion	0.204 ± 0.137	0.059 ± 0.161		Motion	0.209 ± 0.095	0.509 ± 0.191
	Video	0.234 ± 0.075	− 0.485 ± 0.218		Video	0.156 ± 0.085	0.798 ± 0.256

#### Skill assessment predictor performance

As summarised in Table 2, for both criteria of $f_\mathrm{skill}^\mathrm{acc}$ and $f_\mathrm{skill}^\mathrm{pos}$, the skill assessment predictors using both motion and video as the input achieved the highest accuracy, which indicates that using complementary multi-modality data significantly improved performance on $f_\mathrm{skill}^\mathrm{acc}$ (both *p* values < 0.01, paired *t* test at $\alpha =0.05$), while using video input alone can predict $f_\mathrm{skill}^\mathrm{pos}$ well.

Table 3 summarises the results with controlled specificity values by different cut-off values. The performance of the skill assessment predictors, therefore, demonstrates the accuracy with a controlled false positive rate which is critical in many use cases for the skill assessment predictors. Table 4 summarises the ablation results using different window sizes for the two criteria. For $f_\mathrm{skill}^\mathrm{acc}$, the RMSEs decrease as the window size increases, with a decreasing PCC. Notably for predicting $f_\mathrm{skill}^\mathrm{pos}$, the RMSEs with window size 4 are significantly lower than others (*p* values < 0.01), on both data splits A and B.

### The abdominal circumference task

#### Task model performance

The performance of the AC task model on two dataset splits is presented in Table 1. Different from the HC task reported in Section “Task model performance”, we observed that the accuracy of the AC task model decreases as the allowed percentage error $\delta _\mathrm{min}$ increases. This might be because there are more anatomical landmarks required to determine the AC plane than that of HC. A higher $\delta _\mathrm{min}$ may result in higher increased variance in the positive class of the AC task and perhaps more challenging cases too, compared with those in the HC task.

#### Skill assessment predictor performance

The performance of two skill assessment predictors for the AC task is presented in Table 5. The RMSEs generally decrease as $\delta _\mathrm{min}$ increases for $f_\mathrm{skill}^\mathrm{acc}$. A slight increase in RMSE was also observable for $f_\mathrm{skill}^\mathrm{acc}$, perhaps a consequence of the above-discussed biased prediction from the task models.

**Table 3:** Ablation study results of $f_\mathrm{skill}^\mathrm{acc}$, with different cut-off values, at $\delta _\mathrm{min}=0.04$

Split	Cut off	Specificity	RMSE	PCC	Split	Cut off	Specificity	RMSE	PCC
A	0.710	0.8	0.272 ± 0.185	− 0.543 ± 0.323	B	0.702	0.8	0.318 ± 0.257	− 0.017 ± 0.296
	0.799	0.9	0.457 ± 0.197	− 0.076 ± 0.165		0.713	0.9	0.474 ± 0.106	0.023 ± 0.310

**Table 4:** Ablation study results of $f_\mathrm{skill}^\mathrm{acc}$ and $f_\mathrm{skill}^\mathrm{pos}$, with different $\tau$ values, at $\delta _\mathrm{min}=0.02$

Criterion	$\tau$	RMSE	PCC	Criterion	$\tau$	RMSE	PCC
$f_\mathrm{skill}^\mathrm{acc}$	15	0.359 ± 0.146	0.373 ± 0.333	$f_\mathrm{skill}^\mathrm{pos}$	1	0.295 ± 0.111	0.457 ± 0.204
	30	0.328 ± 0.159	0.459 ± 0.397		4	0.229 ± 0.090	0.510 ± 0.185
	60	0.262 ± 0.133	0.278 ± 0.327		8	0.240 ± 0.096	0.294 ± 0.223
	120	0.230 ± 0.156	0.097 ± 0.332		16	0.266 ± 0.135	0.415 ± 0.193

**Table 5:** Performance of skill assessment predictor for the AC task

Criterion	Split	$\delta _\mathrm{min}$	RMSE	PCC	Criterion	Split	$\delta _\mathrm{min}$	RMSE	PCC
$f_\mathrm{skill}^\mathrm{acc}$	C	0.01	0.299 ± 0.075	−0.602 ± 0.326	$f_\mathrm{skill}^\mathrm{pos}$	C	0.01	0.316 ± 0.046	− 0.214 ± 0.179
		0.02	0.294 ± 0.09	− 0.383 ± 0.331			0.02	0.456 ± 0.123	0.374 ± 0.170
		0.03	0.380 ± 0.128	0.147 ± 0.490			0.03	0.486 ± 0.166	0.253 ± 0.257
		0.04	0.389 ± 0.180	0.124 ± 0.382			0.04	0.260 ± 0.099	0.252 ± 0.168
	D	0.01	0.371 ± 0.074	0.401 ± 0.166		D	0.01	0.258 ± 0.066	0.092 ± 0.151
		0.02	0.374 ± 0.076	− 0.421 ± 0.299			0.02	0.458 ± 0.146	0.310 ± 0.182
		0.03	0.363 ± 0.127	0.277 ± 0.444			0.03	0.359 ± 0.133	0.163 ± 0.294
		0.04	0.397 ± 0.180	0.145 ± 0.448			0.04	0.312 ± 0.096	0.124 ± 0.267

**Fig. 2:**
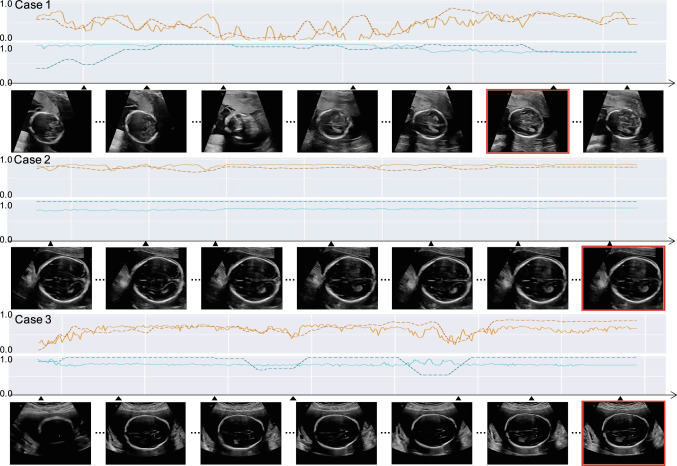
Three example scan clips plotted along the time, with the time-synchronised skill assessment scores, $f_\mathrm{skill}^\mathrm{pos}$ in orange and $f_\mathrm{skill}^\mathrm{acc}$ in blue, with both task model-generated scores (dotted lines) and the predicted scores (solid lines). The red boxed frames were the manually annotated ground truth for the diagnostic planes

### Case studies: comparison with operator experience

With the above-summarised quantitative results, three real-world cases are included in Fig. 2 to demonstrate the clinical relevance and potential use scenarios for these developed skill assessment predictors. More cases are included in the supplementary materials for further reference. *Case 1* was performed by an operator with 7 years of experience. Scores reflect a visible discrepancy in the middle part of the clip, where $f_\mathrm{skill}^\mathrm{pos}$ indicates a low possibility of positive class and $f_\mathrm{skill}^\mathrm{acc}$ still retain a high accuracy of the task; *Case 2* was scanned by a newly qualified operator, yet measured stable and relatively high scores for both criteria during the tested period; *Case 3* was assessing an operator with 6 years of experience, as an example of satisfactory prediction from the skill assessment predictors for both criteria.

## Discussion and conclusion

It is important to note that, although the mixed label generation was found highly effective with available intra-subject manual labels (section “Quality of the mixed label generation”), it may not be able to replace the frame classifiers for generalising to unseen new subjects. These classifiers are example task models in the proposed skill assessment framework, potentially applicable to a wide range of clinical tasks.

This work compared the proposed skill assessment criteria with the operator experience. Section “Results” presented our first proof-of-concept results using clinical data, demonstrating that it is feasible to predict the task model performance and for assessing operator skill. The performance of the skill assessment predictors may be further improved with data acquired during task model-assisted procedures, albeit with potential ethical challenges. Future work will explore the benefits of training the skill assessment predictors simultaneously with the task models, similar to previously proposed image quality assessment [9, 22]. Investigating the robustness of the proposed method with respect to data acquired from different image settings would also be interesting.

In summary, this work first identified a need for new skill criteria when clinical tasks are assisted by machine learning models and, in turn, proposed to automatically predict the skill levels based on task model performance. The experimental results show that the two new skill assessment scores not only are feasible to predict, but also provide informative criteria that are different from existing experience-based metrics.

## Supplementary Information

Below is the link to the electronic supplementary material.

**Supplementary information** This manuscript has one accompanying supplementary file. (5,811 KB)
