# Supplementary material for: Task model-specific operator skill assessment in routine fetal ultrasound scanning
Source: Int J Comput Assist Radiol Surg. 2022 May 12;17(8):1437–44. doi: 10.1007/s11548-022-02642-y (PMC9307537; doi:10.1007/s11548-022-02642-y)
Supplement: Supplementary file 1 — Supplementary information This manuscript has one accompanying supplementary file. (5,811 KB) [file 11548_2022_2642_MOESM1_ESM.pdf]

Supplementary Materials for Task Model-Specific Operator Skill Assessment in Routine Fetal Ultrasound Scanning

Yipei Wang[1*], Qianye Yang[2], Lior Drukker[3], Aris Papageorghiou[3], Yipeng Hu[1,2] and J. Alison Noble[1]

[1*]Institute of Biomedical Engineering, Department of Engineering Science, University of Oxford, Oxford, UK.
[2]Department of Medical Physics and Biomedical Engineering, University College London, London, UK.
[3]Nuffield Department of Women's & Reproductive Health, University of Oxford, Oxford, UK.

*Corresponding author(s). E-mail(s): yipei.wang@eng.ox.ac.uk;

Table 1 Summary of data contribution from the 11 sonographers (S1-S11) used in this study. HC=head circumference, AC=abdominal circumference.

Sonographer		S1	S2	S3	S4	S5	S6	S7	S8	S9	S10	S11	Sum
HC	#Clips	83	11	5	2	1	3	3	1	8	28	1	146
	#Frames	26211	3237	1529	568	212	990	1080	336	2376	9430	360	46329
AC	#Clips	84	7	2	3	0	4	3	0	14	28	4	149
	#Frames	27401	2022	561	930	0	1148	932	0	4394	9021	610	47019

Table 2 Dataset partitioning used in the experiments. HC=head circumference, AC=abdominal circumference, S1-S11 are the 11 sonographers.

Task	Dataset	Development set					Test set							
		Operator				#Clips	Operator							#Clips
HC	A	S1	S2	S9	S10	130	S3	S4	S5	S6	S7	S8	S11	16
	B	S2	S6	S9	S10	50	S1	S3	S4	S5	S7	S8	S11	96
AC	C	S1	S3	S9	S10	128	S2	S4	S6	S7	S11	-	-	21
	D	S2	S3	S9	S10	51	S1	S3	S4	S6	S7	-	-	98

Table 3 Ablation study results for different network architectures at δ_{min}=0.04.

Criterion	δ_{min}	Model	RMSE	PCC
f_{skill}^{acc}	0.01	VGG16	0.380±0.132	0.711±0.366
		ResNet18	0.391±0.132	0.498±0.276
	0.02	VGG16	0.328±0.159	0.459±0.397
		ResNet18	0.305±0.169	0.328±0.447
	0.03	VGG16	0.341±0.239	-0.033±0.247
		ResNet18	0.335±0.249	0.141±0.229
	0.04	VGG16	0.164±0.189	-0.176±0.200
		ResNet18	0.164±0.196	-0.450±0.246

Criterion	δ_{min}	Model	RMSE	PCC
f_{skill}^{pos}	0.01	VGG16	0.246±0.065	0.759±0.188
		ResNet18	0.167±0.036	0.886±0.202
	0.02	VGG16	0.229±0.090	0.510±0.183
		ResNet18	0.247±0.073	0.611±0.231
	0.03	VGG16	0.206±0.063	0.630±0.326
		ResNet18	0.245±0.076	0.513±0.316
	0.04	VGG16	0.171±0.116	0.212±0.257
		ResNet18	0.119±0.063	0.773±0.304

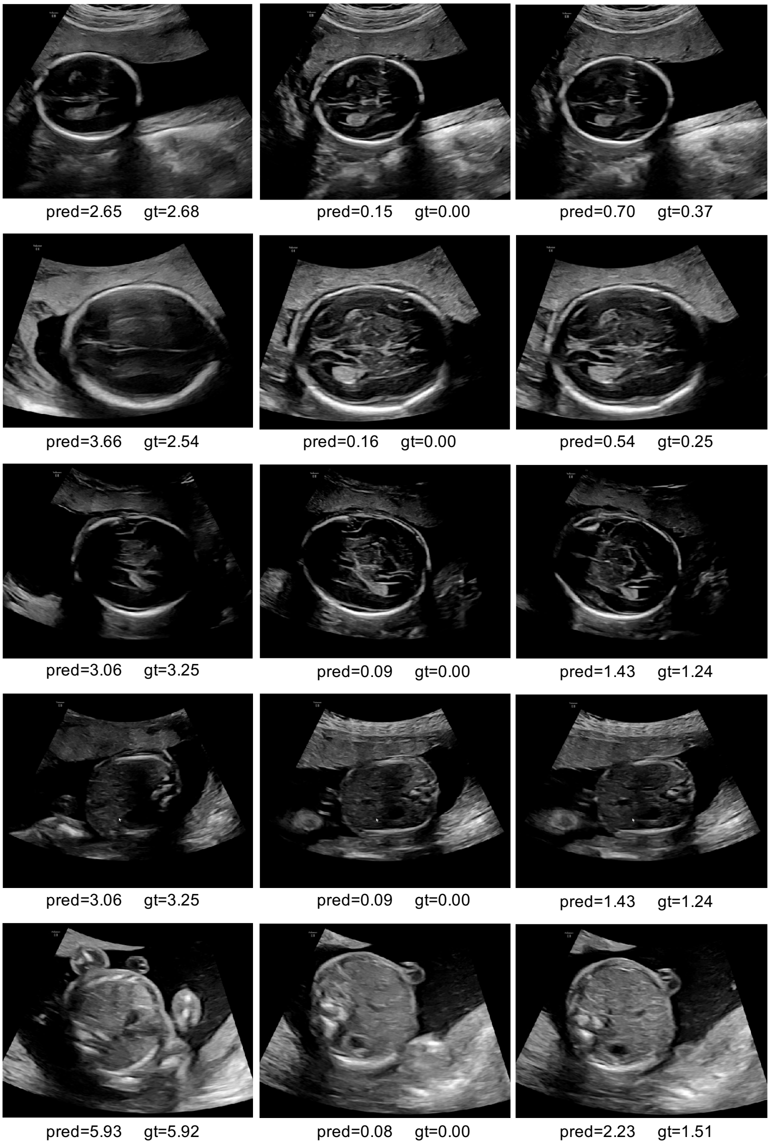

Fig. 1 Visual results of the mixed label generator f_{linear} performance. All images in the same row are from the same scanning sequence and, for reference, the middle images are the diagnostic planes selected by sonographers. Both the predicted percentage relative error $\hat{\delta}$ and the ground-truth percentage error δ are presented.

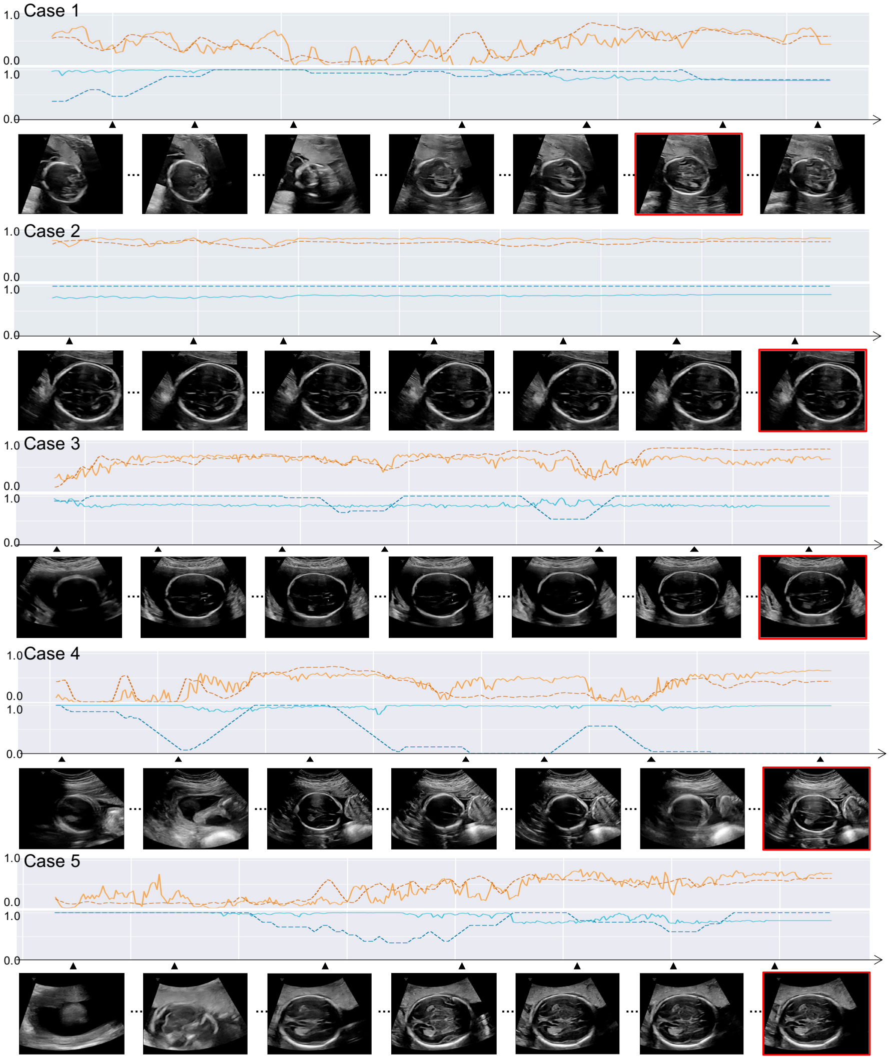

Fig. 2 Additional cases complementing the Section 4.4 and Figure 2 in the main paper. f_{skill}^{pos} and f_{skill}^{acc} are plotted in orange and blue, respectively, with both task model-generated scores and the predicted scores in dotted and solid lines, respectively. The red boxed frames were the manually identified standard planes.
